# Clinical Manifestations of Patients with Coronavirus Disease 2019 (COVID-19) in a Referral Center in Iran

**Published:** 2020-11

**Authors:** Parvaneh Baghaei, Seyed Alireza Nadji, Majid Marjani, Afshin Moniri, Seyed Mohammadreza Hashemian, Hakimeh Sheikhzade, Zahra Abtahian, Jalal Heshmatnia, Atefeh Abedini, Hamidreza Jamaati, Babak Mansourafshar, Zargham Hossein Ahmadi, Mojtaba Mokhber Dezfuli, Sharareh Seifi, Mohsen Sadeghi, Maryam Sadat Mirenayat, Fatemeh Yassari, Somayeh Lookzadeh, Behrooz Farzanegan, Arda Kiani, Maryam Vasheghani, Farzaneh Dastan, Alireza Eslaminejad, Majid Malekmohammad, Mohammad Varahram, Alireza Zali, Payam Tabarsi, Ali Akbar Velayati

**Affiliations:** 1 Clinical Tuberculosis and Epidemiology Research Center, National Research Institute of Tuberculosis and Lung Diseases (NRITLD), Shahid Beheshti University of Medical Sciences, Tehran, Iran,; 2 Virology Research Center, NRITLD, Shahid Beheshti University of Medical Sciences, Tehran, Iran,; 3 Chronic Respiratory Disease Research Center, NRITLD, Shahid Beheshti University of Medical Sciences, Tehran, Iran,; 4 National Research Institute of Tuberculosis and Lung Diseases, Shahid Beheshti University of Medical Sciences, Tehran, Iran,; 5 Lung Transplant Research Center, NRITLD, Shahid Beheshti University of Medical Sciences, Tehran, Iran,; 6 Tracheal Diseases Research Center, NRITLD, Shahid Beheshti University of Medical Sciences, Tehran, Iran,; 7 Mycobacteriology Research Center, NRITLD, Shahid Beheshti University of Medical Sciences, Tehran, Iran,; 8 Research Center for Neurosurgery and Functional Nerves, Shahid Beheshti University of Medical Sciences, Tehran, Iran.

**Keywords:** COVID-19, Features, ICU, Outcomes

## Abstract

**Background:**

Following the recent epidemic of coronavirus disease 2019 (COVID-19) in Wuhan, China, a novel betacoronavirus was isolated from two patients in Iran on February 19, 2020. In this study, we aimed to determine the clinical manifestations and outcomes of the first confirmed cases of COVID-19 infection (n=127).

**Materials and Methods:**

This prospective study was conducted on all COVID-19-suspected cases, admitted to Masih Daneshvari Hospital (a designated hospital for COVID-19), Tehran, Iran, since February 19, 2020. All patients were tested for COVID-19, using reverse transcription-polymerase chain reaction (RT-PCR) assay. Data of confirmed cases, including demographic characteristics, clinical features, and outcomes, were collected and compared between three groups of patients, requiring different types of admission (requiring ICU admission, admission to the general ward, and transfer to ICU).

**Results:**

Of 412 suspected cases, with the mean age of 54.1 years (SD=13.4), 127 (31%) were positive for COVID-19. Following the patients’ first visit to the clinic, 115 cases were admitted to the general ward, while ten patients required ICU admission. Due to clinical deterioration in the condition of 25 patients (out of 115 patients), ICU admission was essential. Based on the results, the baseline characteristics of the groups were similar. Patients requiring ICU admission were more likely to have multiorgan involvement (liver involvement, P<0.001; renal involvement, P<0.001; and cardiac involvement, P=0.02), low O_2_ saturation (P<0.001), and lymphopenia (P=0.05). During hospital admission, 21 (16.5%) patients died, while the rest (83.5%) were discharged and followed-up until March 26, 2020. Also, the survival rate of patients, who received immunoglobulin, was higher than other patients (60.87% vs. 39.13%).

**Conclusion:**

The mortality rate of COVID-19 patients was considerable in our study. Based on the present results, this infection can cause multiorgan damage. Therefore, intensive monitoring of these patients needs to be considered.

## INTRODUCTION

Following a new coronavirus outbreak in China in 2019, many countries have reported an epidemic caused by this virus. So far, more than 1,098,434 confirmed cases of coronavirus disease 2019 (COVID-19) have been reported around the world, with a mortality rate of 59,160 (1). Two previous betacoronavirus epidemics, caused by severe acute respiratory syndrome-related coronavirus (SARSCoV) and the Middle East respiratory syndrome-related coronavirus (MERS-CoV), accounted for 10,000 cases of infection and 1600 deaths. The new coronavirus (SARSCoV-2) has spread rapidly around the world, causing severe pneumonia, acute respiratory distress syndrome (ARDS), multiorgan failure, and death (2).

In Iran, the first case of COVID-19 was recorded in Qom. This city is in close proximity to Tehran, the capital of Iran. Therefore, suspected cases in this city were referred to the National Research Institute of Tuberculosis and Lung Diseases (NRITLD) of Masih Daneshvari Hospital in Tehran, Iran. This hospital has been designated to suspected cases of COVID-19 since the onset of the outbreak. So far, 53,183 confirmed cases of COVID-19 and 3,294 deaths have been reported in Iran (1). Since there is a scarcity of information regarding the clinical manifestations and outcomes of this new viral infection, in this study, we reported the first cases of laboratory-confirmed COVID-19 (n=127), admitted to our center.

## MATERIALS AND METHODS

### Study design and population

This prospective cohort study was conducted on suspected cases of COVID-19 from February 19 to March 2, 2020. This study included all patients with symptoms of an acute respiratory infection, suggestive of COVID-19 (e.g., fever, dry cough, and shortness of breath), with or without a history of travelling to China or other provinces of Iran (e.g., Qom and Gilan) over the past 14 days (before the onset of symptoms) or close contact with suspected cases. The patients in this study were admitted to Masih Daneshvari Hospital in Tehran, which is a National Research Institute of Tuberculosis and Lung Diseases (NRITLD). During the current epidemic, this hospital was designated as a referral center for suspected cases of COVID-19, based on China’s experience with this infection.

Diagnosis of COVID-19 was established, using real-time reverse transcription-polymerase chain reaction (RTPCR) assay for coronavirus. Considering the COVID-19 outbreak, all hospital personnel were completely trained and provided with personal protective equipment, according to contact, droplet, and airborne precautions, recommended by the World Health Organization (WHO) and Centers for Disease Control and Prevention (CDC). The hospital staff also used eye protection to provide care for the patients at this hospital within a short period (3, 4). It should be noted that we had adequate experience regarding the management of H1N1 influenza and other contagious diseases (5).

First, medical masks were given to all patients in the emergency ward and the waiting room. The patients were then transferred to an examination room and underwent assessments by a nurse and a general physician. A physical examination, including body temperature (°C), oxygen saturation, heart rate, respiratory rate (RR) per minute, and blood pressure, was performed. Following lung auscultation and body examination (if needed due to abdominal discomfort), a nasopharyngeal swab specimen was collected from each patient suspected of COVID-19. The collected specimens were examined for coronavirus strains, including OC43, 229E, NL63, and COVID-19, using a Home-Brew real-time PCR assay kit (targeting *E* gene region on a BioRad CFX96 real-time PCR system) at the virology research center of the hospital (6).

Next, chest imaging (chest X-ray or CT scan if needed) was carried out. According to the patients’ general condition and imaging results, they were transferred to the general ward or intensive care unit (ICU). Some patients were treated in the clinic without hospitalization. They were recommended to use their medications at home, wear a mask, frequently wash their hands, maintain social distancing (distance of at least one meter from others), and improve the airflow at home. On the other hand, the hospitalized patients were first treated with an available anti-flu drug (oseltamivir). Since we did not know the cause of pneumonia, broad-spectrum antibiotics, such as vancomycin and meropenem (1 g intravenously every eight hours), as well as levofloxacin, were prescribed.

The patients received supportive treatments, including antipyretic therapy, serum therapy, and supplemental oxygen, using a nasal cannula or a face mask if needed. Six days after the onset of the epidemic, we added hydroxychloroquine (400 mg, once) and lopinavir/ritonavir (400/100 mg every 12 hours; two tablets by mouth twice daily) to the regimen for five days. In some critical cases (with severe ARDS), we added intravenous immunoglobulin (IVIG), as recommended in another study (7). Moreover, we performed laboratory examinations, including complete blood cell count, liver function tests (alanine aminotransferase [ALT], aspartate aminotransferase [AST], and lactate dehydrogenase [LDH]), serum creatinine (Cr) measurement, and other laboratory tests if necessary.

### Data collection

We collected all medical records, as well as laboratory and radiological findings of patients with positive COVID-19 results. The variables included age, gender, comorbidities (e.g., diabetes, cardiac disease, renal disease, and other diseases), obesity (defined as body mass index [BMI] >30 kg/m^2 ^), smoking status, drug abuse history, duration of symptoms, cough, fever, shortness of breath, myalgia, nausea, vomiting, diarrhea, chest imaging results, body temperature, weight, BMI, O_2_ saturation, and outcomes. The collected data were entered independently in SPSS version 16.00 and double checked (SPSS Inc., Chicago, IL, USA). All records were assessed in terms of completeness, reliability, and precision.

### Data analysis

All demographic and clinical information of the patients were analyzed in SPSS version 16.00 (SPSS Inc., Chicago, IL, USA). The patients were divided into three groups, based on the type of hospital admission determined in the first visit to the clinic and the need for ICU care:

1. General ward group: This group included patients with O_2_ saturation <92% in ambient air, RR >20 bpm, pulse rate >100 bpm, chest X-ray infiltrates, and comorbidities (e.g., diabetes, cardiac disease, renal failure, and other conditions, based on the physician’s clinical decision).
2. ICU group: This group included patients, who visited the clinic with O_2_ saturation <90% (with an oxygen mask), RR >30 bpm, and need for mechanical ventilation and vasopressors.
3. ICU transfer group: This group included patients, who were admitted to the general ward in the first visit to the clinic and then developed severe dyspnea, along with conditions described above.

We compared the characteristics, clinical manifestations, and outcomes of the three groups. Categorical variables were compared, using Chi-square or Fisher’s exact test, and continuous variables were assessed, using ANOVA test. All reported P-values are two-sided and a p-value<0.05 was considered statistically significant.

## RESULTS

There were 412 suspected cases of COVID-19, admitted to Masih Daneshvari Hospital between February 19 and March 2, 2020. Overall, 127 (31%) patients, with the median age of 55 years (interquartile range [IQR]: 45–63) were diagnosed with COVID-19 infection. The results showed that most patients were male (76.4%). Also, the majority of patients were Iranian (98.4%). Based on our findings, 12 (9%) infected cases were physicians, 85 (67%) were educated, and 54 (42.5%) had chronic diseases. The history of travelling to other cities with high rates of COVID-19 was reported in 13 patients, and about 42% of patients had a history of close contact with others.

Cough was reported in all patients, followed by fever (n=124), shortness of breath (n=117), myalgia (n=80), nausea (n=21), vomiting (n=20), diarrhea (n=8), and hemoptysis (n=2); the results were similar between the group admitted to the general ward and the group admitted to the ICU. Obesity was reported in 20.5% of patients, diabetes in 28%, renal failure in 4%, and heart disease in 23%. Nine patients showed one-sided involvement, while the rest showed two-sided involvement. Moreover, the patients showed liver (n=33), renal (n=15), and cardiac (n=22) involvement. The results showed that 43.55% of patients had lymphopenia (Table 1 and Table 2).

**Table 1.** Characteristics and demographics of patients infected with COVID-19 based on kind of admission

**Characteristics**	Kind of admission^*^			**P value**

	**Ward admitted (n=92)**	**ICU admitted (n=10)**	IC after admission^*^(n=25)	
**Age, years**	53± 13.9	58.8±8.1	56.1± 12.8	0.30
**Male gender**	72 (78.3)	8 (80)	17 (68)	0.54
**Close contact**	37 (40.2)	4 (40)	12 (48)	0.78
**Travelling history**	7 (7.6)	3 (30)	3 (12)	0.08
**Job**				0.98
**Health worker**	10 (11)	1 (10)	3 (12)	
**Other**	81 (89)	10 (90)	22 (88)	
**Co-disease**	35 (38)	5 (50)	14 (56)	0.24
**DM**	21 (22.8)	5 (50)	9 (36)	0.11
**CRF**	3 (3.3)	0 (0)	2 (8)	0.45
**Obesity**	17 (18.5)	2 (20)	7 (28)	0.58
**Duration of hospitalization**	8± 4	10± 5	9± 5	0.46
**Duration of symptoms**	6± 3	7± 3	7± 2	0.19
**Outcome**				
**Cure**	89 (96.7)	7 (70)	10 (40)	<0.001
**Death**	3 (3.3)	3 (30)	15 (60)	

* Patients who needed intensive care (IC) after hospitalization not at the beginning of admission

Data are n (%) except age (mean± SD), duration of hospitalization and symptoms (days; mean ±SD). DM=diabetes mellitus. CRF=chronic renal failure.

**Table 2.** Clinical and laboratory findings of patients infected with COVID-19 based on kind of admission

**Characteristics**	**Kind of admission**			**P value**

	**Ward admitted (n=92)**	**ICU admitted (n=10)**	**IC after admission (n=25)**	
**Temperature (ºC)**	38.3± 0.8	38.1± 0.7	38.7±0.8	0.09
**BMI (n=82)**	27± 4.5 (n=61)	27.5± 4.6 (n=5)	28.3± 4.8 (n=16)	0.60
**O2Saturation (%)**	89± 5	86± 8	77± 12	<0.001
**Lymphocyte count^¥^**	1245	878	1020	0.26
**Lymphopenia**	36 (39.1)	7 (70)	15 (60)	0.05
**AST (IU/Lit)**	40± 21	57± 30	70± 50	<0.001
**ALT (IU/Lit)**	37± 33	37± 16	46± 38	0.45
**Creatinine (mg/dl)**	1.26± 0.7	1.40± 0.7	1.6± 1.3	0.10
**Liver involvement**	13 (14.1)	4 (40)	16 (64)	<0.001
**Renal involvement**	4 (4.3)	2 (20)	9 (36)	<0.001
**Heart involvement**	12 (13)	1 (10)	9 (36)	0.02
**Chest imaging**				0.11
**Unilateral**	7 (7.6)	2 (20)	-	
**Bilateral**	85 (92.4)	8 (80)	25 (100)	

¥ Median is counted (Cells/μl).

Data are n (%), unless specified otherwise (mean± SD). BMI=body mass index.

AST= aspartate aminotransferase. ALT= alanine aminotransferase.

**Figure 1.**
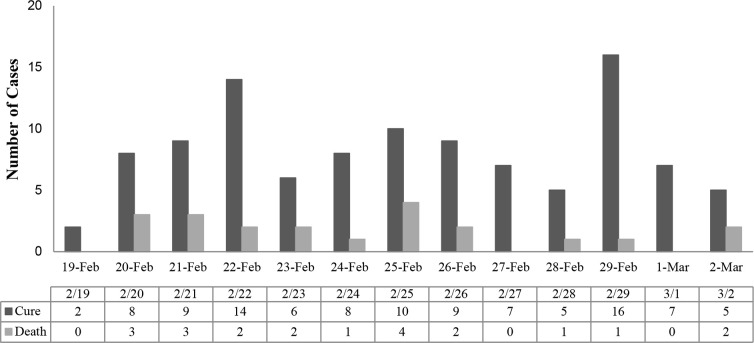
Frequency of number of cured and death patients during 15 days

Overall, 21 (16.5%) patients died during hospitalization. The rest of the patients were discharged with a good general condition to continue their recovery at home. The frequency of cured and deceased cases per day is shown in Figure 1. The incidence of mortality during hospitalization was higher in patients with ICU admission (OR: 31.41, P≤0.001), chronic renal failure (OR: 8.67, P=0.03), liver involvement (OR: 7, P≤0.001), renal involvement (OR: 18.4, P≤0·001), cardiac involvement (OR: 5.36, P=0.001), and not IVIG administration (OR: 4.93, P=0.001) (Table 3). Also, patients receiving IVIG were more likely to survive (61% vs. 39%).

**Table 3.** Characteristics and clinical findings of patients with COVID-19 infected based on outcome

**Characteristics**	**Overall N=127**	**Outcome**		**P value**

		**Survived(n=106)**	**Death (n=21)**	
**Age, years**	54.1±13.4	53.1± 13.6	59.1± 11.3	0.06
**Male gender**	97 (76.4)	82 (77.4)	15 (71.4)	0.56
**Co-disease**	54 (42.5)	42 (39.6)	12 (57.1)	0.14
**DM**	35 (27.6)	27 (25.5)	8 (38.1)	0.24
**CRF**	5 (3.9)	2 (1.9)	3 (14.3)	0.03
**Obesity**	26 (20.5)	22 (20.8)	4 (19)	1
**O2saturation (%)**	87± 8	89± 6	70± 11	<0.001
**Lymphocyte (cells/μl)**	1173	1198	928	0.05
**AST (IU/Lit)**	37.5	37	46	0.01
**Creatinine(mg/dl)**	1.35± 0.83	1.30± 0.76	1.42± 0.34	0.01
**Liver involvement**	33 (26)	20 (18.9)	13 (61.9)	<0.001
**Renal involvement**	15 (11.8)	5 (4.7)	10 (47.6)	<0.001
**Heart involvement**	22 (17.3)	13 (12.3)	9 (42.9)	0.001
**Chest imaging**				
**Unilateral**	9 (7.1)	9 (8.5)	-	0.35
**Bilateral**	118 (92.9)	97 (91.5)	21 (100)	
**ICU admitted**	35 (27.6)	17 (16)	18 (85.7)	<0.001
**IVIG receiving**	23 (18.1)	14 (13.2)	9 (42.9)	0.001

Data are n (%) except age (mean± SD). DM=diabetes mellitus. CRF=chronic renal failure. AST= aspartate aminotransferase. ICU= intensive care unit. IvIg=intra venous immunoglobulin

## DISCUSSION

The results of the present study conducted in Iran showed that among 127 patients with confirmed COVID-19 infection in our hospital, more than 16% died, and 27.6% required ICU admission. There were some differences between the three groups regarding the need for ICU admission, lymphopenia, organ involvement (liver, renal, and cardiac involvement), AST level (higher in patients), and lymphocyte count (lower in patients).

In China, the number of deaths since the onset of COVID-19 (25 deaths in 835 laboratory-confirmed cases) (8) until now (Mar 24, 2020) has been estimated at 3277. Evidence shows that mortality due to this infection is rising rapidly around the world, and the overall case fatality rate was reported to be 2.3%. The elderly and patients with comorbidities, such as cardiovascular disease, diabetes, chronic respiratory disease, hypertension, and malignancy, were found to be at higher risks, respectively (9). In the present study, the rate of mortality was higher than the rate reported by Wu et al. (16% vs. 2.3%) and similar to the rate reported in another similar study (15%) (8).

In the present study, 42.5% of patients had comorbidities, which showed no significant relationship with ICU admission or mortality; nonetheless, patients with chronic renal failure had a higher mortality rate (P=0.03). On the other hand, some patients in our study showed multiorgan involvement, which was associated with a high mortality rate and ICU admission. Therefore, it is necessary to evaluate multiorgan damage as a predictor of mortality (2 , 10, 11). Ongoing evidence suggests human transmission of COVID-19 (12). The first case of COVID-19 in Iran was reported in Qom, a city located near Tehran. Travelling and commuting between these two cities might have led to the transmission of this disease, causing many infections over a short period in Tehran and other cities. In the current study, 13 and 53 patients had a history of travelling and close contact with an infected case, respectively.

Since there is no effective antiviral treatment for COVID-19, patients are being treated with the available drugs in Iran, such as combination of lopinavir and ritonavir with hydroxychloroquine and IVIG in critical cases. The recovery rate in the present study was estimated at 83.5%; so far, 8,913 out of 24,811 confirmed cases have recovered. It has been shown that chloroquine can prevent the replication of several viruses and may function effectively against COVID-19 infection (13–16). Some studies have used a combination of lopinavir and ritonavir, with or without interferon beta-1b or remdesivir, similar to the treatment of SARS-CoV and MERS-CoV; however, the efficacy of these drugs for COVID-19 is unknown (17–19). The first case of COVID-19 in the United States was treated by remdesivir (20). It should be noted that the COVID-19 outbreak occurred right before the Iranian New Year holiday (March 20), when people go shopping in crowded streets and malls, visit their families, and participate in many traditional ceremonies. Therefore, management of this epidemic has been quite challenging in Iran.

In conclusion, this study showed that multiorgan involvement could be a predictor of ICU admission and high fatality rate in COVID-19 cases. Also, IVIG administration can be considered as a supportive treatment for COVID-19 to increase the recovery rate. Further research and reports from other countries can provide more comprehensive information about this disease to improve monitoring and treatment.
